# The association of perceived ethnic discrimination and institutional verbal violence with chronic stress in an immigrant sample: The role of protective factors - results from the VIOLIN study

**DOI:** 10.1016/j.jmh.2024.100260

**Published:** 2024-08-04

**Authors:** Felicitas Hauck, Andrea Borho, Lucía Romero Gibu, Mojib Atal, Sevil Dederer, Petra Bendel, Eva Morawa, Yesim Erim, Silke Jansen, Nicolas Rohleder

**Affiliations:** a Department of Psychology, Friedrich-Alexander-University Erlangen-Nürnberg, Nägelsbachstraße 49a, 91052 Erlangen, Germany; b Department of Psychosomatic Medicine and Psychotherapy, University Hospital of Erlangen, Friedrich-Alexander University Erlangen-Nürnberg, Hartmannstraße 14, 91052 Erlangen, Germany; c Department of Romance Studies, Friedrich-Alexander-University Erlangen-Nürnberg, Bismarkstraße 1, 91054 Erlangen, Germany; d Institute of Political Science, Friedrich-Alexander-University Erlangen-Nürnberg, Kochstraße 4, 91054 Erlangen, Germany

**Keywords:** Ethnic discrimination, Verbal violence, Chronic stress, Self-compassion, Migration generation, Citizenship status

## Abstract

Immigrants are exposed to a variety of stressors, such as ethnic discrimination, and therefore experience a higher risk of developing adverse health outcomes. However, the role of potentially protective psychological factors is not well-studied. The present study addresses the question how discrimination and institutional verbal violence (IVV)^1^ are associated with chronic stress in an immigrant sample. In addition, this study highlights moderating effects of migration-specific variables (first or second migration generation and citizenship status).

Participants (*n* = 232; 69.4 % female) completed an online-survey, which included demographics, questionnaires (Everyday Discrimination Scale, EDS; Perceived Stress Scale, PSS-4; Resilience Scale, RS-11; Self-Compassion Scale, SCS-SF) as well as a self-developed questionnaire on institutional verbal violence. Only participants living in Germany with migration background (self or one parent migrated to Germany) were included.

Results showed that perceived discrimination and institutional verbal violence were highly associated with chronic stress. Further, self-compassion buffered the connection between discrimination and stress, whereas resilience was no protective factor. The inclusion of migration-specific variables showed that the second-generation sub-group experienced less discrimination-related stress and self-compassion was shown to be particularly protective within this sub-group. Citizenship status did not appear to be a moderator, but especially persons with temporary or permanent residence status, compared to German/EU-citizens, reported higher values of verbal violence and discrimination-related stress.

These findings highlight the importance of considering not only psychological but also structural and societal protective and risk factors, as they may be differentially associated with immigrants’ stress perceptions. Implications for future research and practical implementations are presented.

## 1 Introduction

Some persons face severe forms of stressors due to their perceived group affiliation, for example members of minorities and immigrants. Focusing on migration, various forms of stressors must be considered, which may occur before, during or after the actual migration process (Erim and Morawa, 2016; Demiralay and Haasen, 2011; Mak et al., 2021; Berry et al., 1987). Experiencing these stressors can influence physical and mental health negatively (Aichberger et al., 2012; Bermejo et al., 2010; Mewes et al., 2017). Still, study results are inconsistent and show the need for continuing research in this field (Glaesmer et al., 2009; Mewes et al., 2010). Experiencing chronic stress, due to exposure to repeated stressors or such with high intensity, may lead to adverse outcomes. This is explained by the *Allostatic Load Model* (McEwen, 1998): underlying physiological pathways promote the development of diseases, for example by fostering a dysregulation of the inflammatory system and thereby triggering pathophysiological processes (Rohleder, 2019).

One particularly detrimental post-migratory stressor is ethnic discrimination. It generates stress as individuals experience unfair treatment due to their (perceived) group membership (Clark et al., 1999; Williams and Mohammed, 2009). Such experiences happen unpredictably and uncontrollably, and are therefore difficult to cope with (Pascoe and Smart Richman, 2009). Previous research shows that ethnic discrimination promotes the development of mental health problems or illnesses, e.g. depressive and anxiety symptoms (Pascoe and Smart Richman, 2009; Straiton et al., 2019), suicidal thoughts (Coimbra et al., 2022), has negative impact on psychological well-being (e.g., self-esteem, life-satisfaction), as well as leads to increased perceived stress (Schmitt et al., 2014; Goreis et al., 2020).

Contexts that may amplify and worsen the negative effects of discrimination upon immigrants’ health are institutional settings. Previous research found that institutional interactions are shaped by unequal power distributions due to knowledge differences and dependencies (e.g., imagining an immigrant person in a Foreigner's Registration Office) (Kauff et al., 2017). These, as well as formalized interaction scripts may shape the communication negatively: One interaction partner probably enters the conversation rather involuntary and both have different aims within the situation. Thus, general asymmetries between both persons based upon differing interests and assumptions aggravate the interaction (Jansen and Romero Gibu, 2021; Rosenberg, 2014; Porila and Jan, 2008). To a help-seeking client, an institution or its representative may seem to represent the society as a whole, which subsequently may lead to the perception of overall rejection thereof when experiencing discrimination (Kauff et al., 2017).

In institutional contexts, predominantly subtle, covert forms of violent behaviour may appear, such as verbal forms, which may be more likely to be tolerated as they seem legitimized by the inherent power imbalance (Kauff et al., 2017; Jansen and Romero Gibu, 2021). Verbal violence (VV), a concept originating from linguistics, is defined as “linguistic behaviour with detrimental effects on the interlocutor” (Jansen and Romero Gibu, 2021). It should be noted that the concept of discrimination has certain overlaps with VV, without being identical to it. Institutional contexts may facilitate the development of VV, as the reduction of an individual's interactional scope is already inherent to the situation (Jansen and Romero Gibu, 2021; Bonacchi, 2017). While verbal forms of institutional discrimination are scarcely researched and institutional verbal violence (IVV) has not yet been quantified, some studies have revealed particularly detrimental health effects resulting from discriminatory behaviour of authority members or state representatives against minority group members. For example (Kalinowski et al., 2022) found that African American women who experienced police discrimination, such as being unfairly stopped, searched or questioned, reported higher levels of depressive symptoms. Further, in a study with European adolescents, discrimination by the police and security personnel had the strongest negative impact on self-reported psychosomatic health (Kauff et al., 2017). Besides these institutional forms of discrimination, further structural and context factors, such as country-specific immigration laws and immigration history may intensify or mitigate migration or discrimination-related adverse health outcomes. Especially when looking at IVV, it is important to consider the receiving country's political situation, as depending on residence status and residence permits, interactions in institutions may vary for different immigrant groups. Tsuchiya et al. (2023) showed that individuals without citizenship status reported worse self-rated health compared to individuals with valid citizenship status, especially when experiencing high levels of ethnic discrimination. Further migration-specific variables may also contribute to chronic and acculturative stress and therefore condition the health status of immigrants, including generation of migration (Abdulhamed et al., 2022; Cano et al., 2021; Morawa et al., 2017).

The previous section emphasized the harmful potential for detrimental effects of structural, institutional and societal factors in particular. However, besides structural approaches, also psychological factors and personal characteristics need to be taken into account when analysing discrimination-related health disparities. These are often highlighted when focusing on health promotion, as they can serve as starting points for interventions on an individual level, but also provide valuable insights for public policies and service providers (Cano et al., 2023). One concept that has been well studied in this context is resilience. It is defined as a process of successful adaption to difficult life circumstances and disruptions while maintaining psychological and physical functioning (Connor and Davidson, 2003; Bonanno, 2004), and leading a better life beyond circumstances' predictive bounds (Troy et al., 2023). In summary, it may be described as an overarching ability that keeps individuals healthy even though they are facing hardships and difficulties; a form of psychological resistance (Schumacher et al., 2004). Past studies predominantly focused on specific protective factors and barely examined the direct operationalization of resilience and its interaction between discrimination and health. However, results are inconsistent. Some found buffering, positive effects of resilience upon psychological distress while facing discrimination (Wu et al., 2021), or negative associations of racial discrimination and resilience (Brown and Tylka, 2011), whereas others did not find specific protective effects of resilience on the relationship between discrimination and self-rated health (Cano et al., 2023).

A specific resilience-promoting factor (Bluth et al., 2018), which may counteract the adverse outcomes of ethnic discrimination is self-compassion. Self-compassion is defined as a compassionate attitude toward oneself and acknowledging difficult feelings as part of being human (Neff, 2003a; 2003b). Further, by turning towards difficult feelings and experiences with kindness and awareness, self-compassion represents an effective emotion regulation strategy. It is applied in various domains, such as stress prevention and psychotherapy (Han and Kim, 2023; Neff and Germer, 2022; Zeller et al., 2015). Previous research showed its positive buffering effects when experiencing acute (Arch et al., 2014; Pace et al., 2009) as well as chronic stress (Zhang et al., 2016; Neff et al., 2007). Self-compassion is a multidimensional construct consisting of six sub-constructs: self-kindness versus self-judgement, humanity versus isolation, mindfulness versus over-identification (Neff, 2003b). In previous discrimination research, this concept and its sub-constructs were often included as moderator variables, mitigating or amplifying the detrimental effects of ethnic discrimination upon health. As a risk factor, the sub-facet self-judgement was found to be a moderator between racial discrimination and anxiety and somatic symptoms (Browne et al., 2022). However, self-kindness served as a protective factor between racial discrimination and depression (Liu et al., 2020). Self-compassion further moderated the link between weight discrimination and psychological distress (Pullmer et al., 2021) and the relationship between experiencing structural discrimination, belonging to a sexual- and/or gender-minority and depressive symptoms (Vigna et al., 2020). Still, its consequences for chronic stress experiences in European research are understudied.

### 1.1 Present study

While it becomes more important to understand factors affecting health and well-being of immigrants, particularly European research assessing structural and psychological factors in the form of risk and protective factors, is scarce. To examine protective effects of self-compassion and resilience, and to further examine influences of structural factors, such as citizenship status, we conducted an online-survey that targeted immigrants living in Germany. Based upon previous research, we developed a theoretical model, in which we consider chronic stress as the primary outcome due to its detrimental effect on health, with IVV and discrimination acting as stressors. Furthermore, the inclusion of the noted risk and protective factors into these models are of interest. Thus, we hypothesized a positive association of IVV and ethnic discrimination with reported chronic stress. We assume that self-compassion and resilience buffer these associations. Migration-specific variables, such as citizenship status and migration generation might serve as risk or protective factors.

## 2 Methods

### 2.1 Procedure and study design

This study was designed as a cross-sectional online-survey in the VIOLIN-project (Verbal Violence in Institutions) at Friedrich-Alexander-Universität (FAU) Erlangen-Nürnberg and consisted of a basic module asking for demographics, migration related variables and several questionnaires, as well as additional modules. The basic module and further questionnaires, unless otherwise described, were translated in nine languages (Pashto, Farsi/Dari, French, Spanish, Turkish, Polish, Arabic, English, German) using the TRAPD-method (The TRAPD method for survey translation, 2018). For the present study, only a fraction of all participants is examined, as not everyone completed the analysed additional module. The survey was presented in an online survey tool (Tivian, Questback) and advertised via social network, newspapers and migrant organisations. Further, the link was sent to befriended research groups and public corporations, such as integration commissioners, integration councils and anti-discrimination bodies. The study received ethical approval by the Ethics Committee of the FAU (protocol: 451_19B). Participants gave informed consent before participating.

### 2.2 Participants

The final sample consisted of 232 participants (69.4 % female) who were on average 39 years old (*SD* = 11.1), ranging from 18 to 76 years. Participants had to be 18 years or older and currently living in Germany. They were only included if they themselves or at least one parent immigrated to Germany, which means oneself or at least one parent was not born in Germany or they or at least one parent was born in Germany as non-Germans. This is consistent with the in Germany widely used term "migration background" (Migration und Integration. Statistisches Bundesamt n.d.). In accordance with previous studies, the equivalent terms first (self and parents are born abroad) and second generation-immigrants (only parents are born abroad) were used in the following (Abdulhamed et al., 2022; Kim et al., 2021; Morawa and Erim, 2018). Thirty-six (15.5 %) participants reported to be born in Germany with both parents being born abroad and were therefore second generation-immigrants. First-generation immigrants were 196 (74.5 %) participants. One participant reported to be born abroad, but having one parent being born in Germany. First generation participants migrated to Germany with a mean age of 9.7 years (*SD* = 33.6). The three most frequently mentioned reasons were for family and partnership reasons (*n* = 54; 23.3 %), to study (*n* = 46; 19.8 %) and flight/forced migration (*n* = 35; 15.1 %).

Overall, participants were born in 42 different countries. The three most frequent countries of birth were Poland (*n* = 26), Mexico (*n* = 20) and Turkey (*n* = 16). The three most frequent countries of birth for participants’ mothers were Turkey (*n* = 34), Poland (*n* = 21) and Mexico (*n* = 20); and Turkey (*n* = 35), Mexico (*n* = 20) and Poland (*n* = 17) for fathers. Regarding citizenships, 115 (49.6 %) participants reported to hold German citizenship, 23 (9.9 %) persons were citizens of a country of the European Union (EU) and thus had the opportunity for permanent residence. Seventy-six (32.8 %) persons reported to have a double citizenship. Twenty-nine (12.5 %) persons reported having applied for asylum. Permanent residence permit was reported by 41 (17.7 %) participants, 41 (17.7 %) participants held temporary residence status. Three (1.3 %) participants reported to have no title (e.g. suspension of the obligation to leave the country, ‘Duldung’).

The language in which participants completed the questionnaire was predominantly German (*n* = 182; 78.4 %) and Spanish (*n* = 31; 13.4 %), only a few completed in English (n = 16; 6.9 %). The most frequently mentioned first language was Spanish (*n* = 90; 38.8 %), followed by German (*n* = 55; 23.7 %) and Turkish (*n* = 24; 10.3 %). Forty-seven (20.3 %) persons claimed to speak two first languages. German language proficiency was self-evaluated according to the Common European Framework of Reference for Languages (CEFR), whereas 17 participants (7.3 %) reported level A proficiency (basic user), 49 (21.1 %) level B (independent user), 111 (47.8 %) level C (proficient user) and 55 (23.7 %) reported to be German native speakers.

Regarding religious affiliation, 84 (36.2 %) participants reported to be Christian. The second most reported religion was Islam (*n* = 56; 24.1 %), 83 (35.8 %) participants reported not to belong to any religion. Regarding relationship status, 54 (23.3 %) participants were single, 36 (15.5 %) were in a relationship, 121 (52.2 %) were married and 21 (9.1 %) were divorced or widowed; 110 (47.4) participants reported to have children. Participants reported that they had gone to school for an average of 12.79 (SD = 3.46) years, 209 (90.1 %) participants finished secondary school with a degree. Currently, 158 (68.1 %) reported to work, of which 99 persons reported to work full-time (62.7 % of persons who reported to work); 25 (10.8 %) persons reported to study and 10 (4.3 %) persons reported to be housewives or –husbands.

### 2.3 Measures

#### 2.3.1 Verbal violence in institutions

The Verbal Violence Questionnaire was developed by results gained from interviews conducted by our research team with immigrants in Germany and their experiences in German institutions (Jansen and Romero Gibu, 2021). These interviews were based on the Critical Incident Technique (Flanagan, 1954) and led to the development of a VIOLIN corpus (Jansen and Romero Gibu, 2021). The developed questionnaire asks for experiences of IVV, concerning interactions, non-verbal communication and language use. The scale was revised through discourses within the research group; the final version consists of 24 items. The items need to be answered on two different response scales: One asks for frequency (“How often?”), the second for intensity (“How bad?”) of an incident. The frequency subscale can be answered with 0 = *never* to 4 = *very often*. The perceived intensity subscale ranges from 0 = *not at all* to 4 = *very*. An example item is: “The person spoke to me very slowly because he/she thought I didn't understand him/her.” This resulted in sum values for frequency and for intensity. To calculate a weighted total score for verbal violence, frequency and intensity were multiplied for each item. These values were then added up and divided by the number of items. This resulted in a range with values from 0 to 16. The internal consistencies for the present study can be considered as excellent (Cronbach's alpha for total score: α = 0.96; frequency subscale: α = 0.94, intensity subscale α = 0.97).

#### 2.3.2 Perceived ethnic discrimination

Perceived, ethnic discrimination was measured with the Everyday Discrimination Scale (EDS) by Williams et al. (Williams et al., 1997). The scale consists of nine items that can be rated on a 6-point response scale (1 = *never* to 6 = *almost every day*), are constructed as questions, which ask for discriminatory events in everyday life (e.g. “Are you treated with less respect than other people?”), with higher values indicating more experiences of perceived discrimination. Cronbach's alpha for the present study can be considered as excellent (α = 0.92).

#### 2.3.3 Chronic stress

The four-item form of the Perceived Stress Scale (PSS-4), originally with 14 items, by Cohen et al. (Cohen et al., 1983) was used to measure chronic stress within the last four weeks. The scale consists of four items (e.g., “In the last month, how often have you felt that you were unable to control the important things in your life?”) with a 5-point response scale (0 = *never* to 4 = *very often*). Validated translation were used for the Spanish version by Remor (Remor, 2006) and the German version by Klein et al. (Klein et al., 2016). An overall acceptable intern validity for this sample was found (Cronbach's α = 0.70).

#### 2.3.4 Self-compassion

To measure self-compassion, the short form of the Self-Compassion Scale (SCS-SF) was used (Neff, 2003b; Raes et al., 2011). The short form consists of 12 items ranging from 1 = *almost never* to 5 = *almost always* (e.g. “When I fail at something important to me I become consumed by feelings of inadequacy”). The overall internal validity for our sample can be considered as good (α = 0.83). The questionnaire was used in its English original form, in the German version by Hupfeld and Ruffieux (Hupfeld and Ruffieux, 2011), as well as in the Spanish version by Garcia-Campayo et al. (Garcia-Campayo et al., 2014).

#### 2.3.5 Resilience

A short form (RS-11) of the Resilience Scale, originally by (Wagnild and Young, 1993), was used to measure the construct of resilience. The short form consists of 11 items and was initially constructed in a German version by (Schumacher et al., 2004). The items can be rated upon a 7-point response scale from 1 = *disagree* to 7 = *agree* (example item: “I usually manage one way or another”), with persons reaching higher values meaning being more resilient. Cronbach's alpha was considered as excellent within the present study (α = 0.91).

#### 2.3.6 Migration-specific stressors

As stressors specific to migration history, first or second generation background and the citizenship status were considered. The variable for citizenship status was recalculated and the categories 1 = *temporary residence status* (combined the categories “no title” and “temporary”), 2 = *permanent residence status*, and 3 = *German citizenship/citizens of another European Union country* were formed.

### 2.4 Analytic strategy

All analyses were conducted with IBM SPSS Statistics 29 (Chicago, Illinois, USA). In the first step of data processing, potential missing values were checked. The SCS-SF was missing for 12 participants, so they had to be excluded for respective analyses. For all questionnaires and their subscales, total scores or mean values were computed. Further, outliers (*z* > 3.29) as well as data distribution were checked. One outlier was found for the age variable, one for the EDS and three for the IVV total score. These values were checked for separately in the respective calculations. Considering data distribution, only the SCS-SF total score was normally distributed, checked with Shapiro-Wilk tests. All other scales and subscales were double-checked with P-P-Plots and their values for skewness and kurtosis. Especially the RS was strongly left-skewed, the IVV total score and the EDS were strongly right-skewed. Due to the large sample, statistical procedures that are robust to normal distribution violation were still used (Field, 2017). For linear and multiple regression analyses, as used in our analyses, other factors are more important for the ideal regression estimate, such as homoscedasticity (Schmidt and Finan, 2018; Hayes, 2022). One participant had to be excluded for several regression analyses due to outlier values checked via standardized and studentized residuals. As all residuals for regression analyses were not normally distributed, bias corrected and accelerated (BCa) bootstrapping with 5000 samples was employed (Field, 2017; Hayes, 2022). Analyses included multiple regression, moderation and moderated moderation models using PROCESS 4.1 (Hayes, 2022). Self-compassion and resilience were used as moderator variables, influencing the connection between perceived ethnic discrimination and chronic stress, as well as IVV and chronic stress (model 1). Further, migration generation and citizenship status were included as moderators in the aforementioned moderation models (model 3). To deal with heteroscedasticity in the moderation models, heteroscedasticity-consistent (HC) standard errors, more precisely HC3 (Davidson-Mackinnon), were used. This method showed the best results to implement robust standard errors in heteroscedasticity when ordinary least squares estimates are applied (Nwangburuka and Nwakuya, 2023). Further, variance inflation factors (VIF) were analysed to control for multicollinearity. As these were rather high (>10), variables were mean-centered for moderation analyses. Overall, results were considered significant with *p* < .05.

## 3 Results

### 3.1 Preliminary analyses

**Fig. 1:**
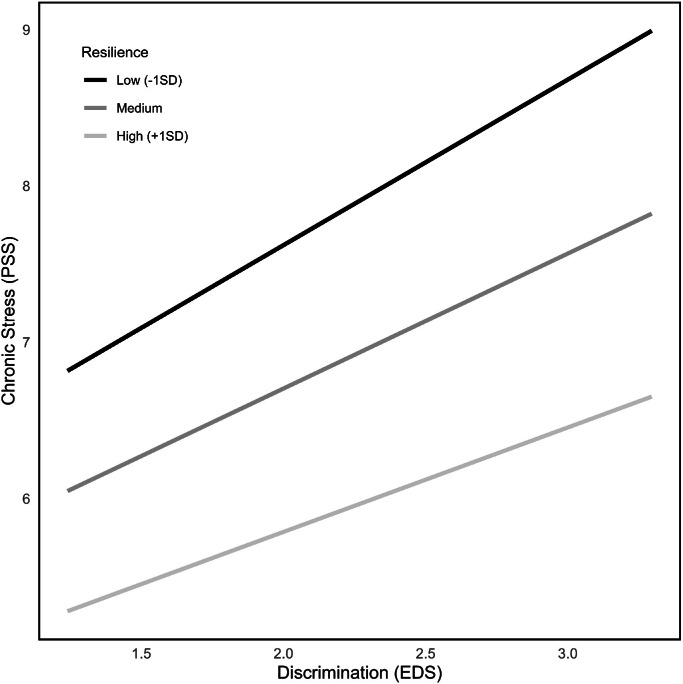
Interactions between PSS and EDS at different levels of resilience.

Intercorrelations are shown in Table 1. The influence of age upon these variables was examined. Further, sex differences for these variables were checked via *t*-tests. Most variables did not differ significantly between men and women, except the IVV total score (*T* = 2.18, *p* = .03) and the IVV frequency score (*T* = 2.81, *p* < .01), with women reporting more IVV. Results for tests of group differences between the first or second generation as well as the citizenship status groups can be viewed in Fig. 1 in the Appendix.

**Table 1:** Zero-order, non-parametric correlations (Spearman), means and standard deviations for relevant variables.

Variable	1	2	3	4	5	6	7	8
1. PSS	–							
2. SCS	−0.40⁎⁎⁎	–						
3. RS	−0.35⁎⁎⁎	.35⁎⁎⁎	–					
4. EDS	.23⁎⁎⁎	−0.10	−0.11	–				
5. IVV-F	.17⁎⁎	−0.09	−0.02	.75⁎⁎⁎	–			
6. IVV-I	.16	−0.04	−0.10	.77⁎⁎⁎	.95⁎⁎⁎	–		
7. IVV sum	.16*	−0.10	−0.02	.73⁎⁎⁎	.97⁎⁎⁎	.97⁎⁎⁎	–	
8. age	−0.12	.25⁎⁎⁎	.20⁎⁎	−0.17⁎⁎	−0.14*	−0.17	−0.15*	–
*M*	6.96	3.20	2.76	2.27	25.46	57.47	2.88	39.01
*SD*	3.23	0.67	1.07	1.02	18.59	35.79	2.89	11.10

*Note.* EDS = Everyday Discrimination Scale; PSS = Perceived Stress Scale; SCS-SF = Self-Compassion Scale – Short Form; RS = Resilience-Scale 11; IVV-*I* = Institutional Verbal Violence Intensity; IVV-*F* = Institutional Verbal Violence Frequency; IVV sum = Institutional Verbal Violence total score; *M* = mean value; *SD* = Standard Deviation.

⁎ *p* < .05 (two-sided).

⁎⁎ *p* < .01 (two-sided).

⁎⁎⁎ *p* < .001 (two-sided).

### 3.2 Regression models: associations between discrimination, IVV and perceived chronic stress

As Table 1 shows, significant positive correlations between ethnic discrimination and chronic stress (*p* < .001), as well as IVV and chronic stress were found (*p* < .001). IVV intensity did not correlate with perceived chronic stress (n.s.), whereas its frequency showed significant associations (*p* = .01).

As a first step, simple regression analyses were calculated to confirm the association between IVV and discrimination with chronic stress. In a second step, a multiple regression analysis was calculated, including both the predictors IVV and perceived ethnic discrimination; chronic stress served as outcome variable. IVV was able to predict chronic stress perceptions significantly (*F*(1229) = 10.15, *p* < .01; *b* = 0.23, BCa 95 % CI [0.07; 0.37]), as well as ethnic discrimination (*F*(1230) = 22.46, *p* < .001; *b* = 0.94, BCa 95 % CI [0.52; 1.35]).

Further, a multiple regression analysis was run, including both aforementioned variables as predictors in one model. The overall model was significant (*F*(2229) = 11.49; *p* < .001) with an R² for the overall model of 0.09 (adjusted R² = 0.08), which indicates that 8 % of the variance in chronic stress was explained by discrimination and IVV. Ethnic discrimination was a robust predictor for chronic stress (*b* = 1.11, BCa 95 % CI [0.42; 1.71]), whereas IVV did not explain any further variance when included in the regression model (*b* = −0.08, BCa 95 % CI [−0.32; 0.18]).

### 3.3 Moderation models with self-compassion and resilience

To analyse potential protective functions upon the association between IVV, discrimination and chronic stress, four moderation models were computed in PROCESS (Hayes, 2022). Self-compassion and resilience were tested as moderator (W), whereas IVV or discrimination were tested as predictor (X) and chronic stress as the outcome (Y).

Analysing the effect of resilience upon IVV and chronic stress in the first step (model 1), results revealed an overall significant model, *F*(3226) = 10.41, *p* < .001, predicting 14.44 % of the variance, but the interaction effect was not significant (n.s.). When including self-compassion instead of resilience into the model (model 2), the overall model was significant (*F*(3216) = 17.06, *p* < .001), explaining 19.20 % of variance; no interaction effect between IVV and self-compassion was found (n.s.).

**Fig. 2:**
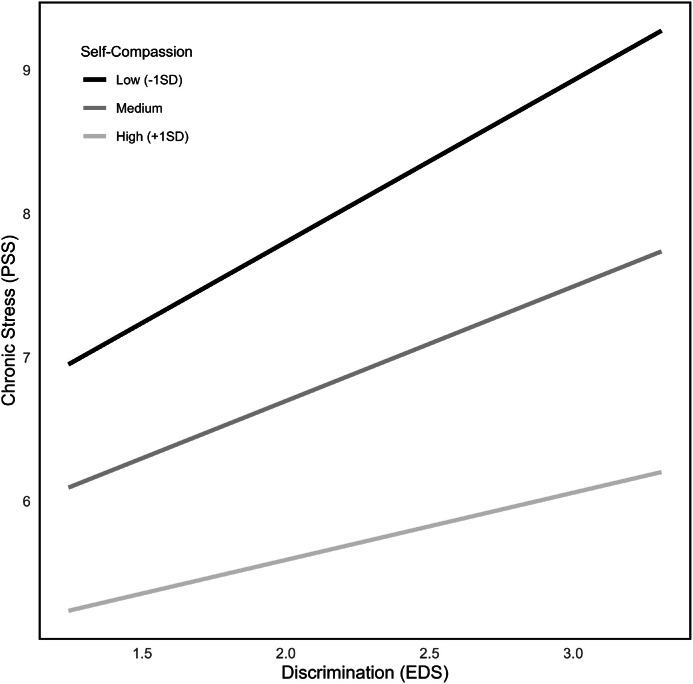
Interactions between PSS and EDS at different levels of self-compassion.

These models were repeated for ethnic discrimination as predictor variable X. For resilience as moderator (model 3), results revealed an overall significant model (*F*(3226) = 13.57, *p* < .001), explaining 18.15 % of variance, but no significant interaction effect was found (n.s.; see Fig. 1). For self-compassion as moderator (model 4), the overall model reached significance (*F*(1216) = 30.72, *p* < .001; see Fig. 2), explaining 24.14 % of variance. Results showed moreover, that self-compassion moderated the effect between ethnic discrimination and chronic stress significantly (ΔR² = 1.2 %, *F*(3216) = 4.39, *p* = .04). Further values of these moderation analyses can be found in Table 2.

**Table 2:** Moderation analyses with EDS and IVV as predictors, self-compassion and resilience as moderator variables and PSS as outcome.

	Coefficient	SE	*t*	*p*	95 % CI	
					Lower	Upper
**Model 1 (IVV and resilience)**						
RS	−0.09	0.02	−4.47	.001	−0.13	−0.05
IVV	0.23	0.08	2.98	.003	0.08	0.38
RS x IVV	−0.01	0.01	−1.31	.19	−0.02	0.00
**Model 2 (IVV and SCS)**						
SCS	−1.88	0.30	−6.21	.001	−2.48	−1.28
IVV	0.19	0.08	2.47	.01	0.04	0.34
SCS x IVV	−0.08	0.11	−0.71	.48	−0.30	0.14
**Model 3 (EDS and resilience)**						
RS	−0.08	0.02	−4.28	.001	−0.12	−0.04
EDS	0.86	0.19	4.52	.001	0.49	1.24
RS x EDS	−0.02	0.01	−1.22	.22	−0.04	0.01
**Model 4 (EDS and SCS)**						
SCS	−1.78	0.28	−6.37	.001	−2.34	−1.23
EDS	0.80	0.18	4.31	.001	0.43	1.16
SCS x EDS	−0.49	0.23	−2.10	.04	−0.95	−0.29

*Note.* SE = Standard Error; *t* = *t*-Value; *p* = *p*-Value; 95 % CI = Confidence Interval; EDS = Everyday Discrimination Scale; SCS = Self-Compassion Scale – Short Form; RS = Resilience-Scale 11; IVV = Institutional Verbal Violence total score; PSS = Perceived Stress Scale.

### 3.4 Moderation models with migration-specific variables

Further, the potential mitigating or buffering effects of migration-specific variables upon chronic stress were examined. Again, model 1 by PROCESS was used (Hayes, 2022), in which migration generation and citizenship status were included as moderator variables. Overall, four models were calculated (see Table 3).

**Table 3:** Moderation analyses with migration-specific variables as moderators and PSS as outcome.

	Coefficient	SE	*t*	*p*	95 % CI	
					Lower	Upper
**Model 5 (IVV and generation)**						
Generation	−0.21	0.60	−0.36	.72	−1.39	0.96
IVV	0.26	0.09	2.98	.003	0.09	0.43
Gen x IVV	−0.30	0.23	−1.30	.19	−0.74	0.15
**Model 6 (IVV and citizenship status)**						
Status W1	−0.99	0.68	−1.47	.14	−2.33	0.34
Status W2	−0.62	0.53	−1.17	.24	−1.66	0.42
IVV	0.52	0.24	2.15	.03	0.04	0.99
Stat W1 x IVV	−0.09	0.28	−0.33	.74	−0.65	0.47
Stat W2 x IVV	−0.45	0.27	−1.69	.09	−0.98	0.07
**Model 7 (EDS and generation)**						
Generation	−0.22	0.56	−0.38	.70	−1.33	0.89
EDS	1.21	0.22	5.55	.001	0.78	1.64
Gen x EDS	−1.44	0.61	−2.35	.02	−2.64	−0.23
**Model 8 (EDS and citizenship status)**						
Status W1	−0.83	0.71	−1.16	.25	−2.24	0.58
Status W2	−0.30	0.56	0.54	.59	−1.40	0.80
EDS	1.19	0.59	2.00	.047	0.02	2.36
Stat W1 x EDS	−0.004	0.73	−0.01	.99	−1.44	1.43
Stat W2 x EDS	−0.45	0.68	−0.66	.51	−1.80	0.90

*Note.* SE = Standard Error; *t* = *t*-Value; *p* = *p*-Value; 95 % CI = Confidence Interval; Gen = Immigration Generation; IVV = Institutional Verbal Violence total score; EDS = Everyday Discrimination Scale; Status/Stat = Citizenship Status (1 = temporary, 2 = permanent; 3 = German or EU citizenship); W1 = Moderator 1 (Coded categorical variable W with 1 = 0; 2 = 1; 3 = 0); W2 = Moderator 2 (coded with 1 = 0; 2 = 0; 3 = 1); PSS = Perceived Stress Scale.

**Fig. 3:**
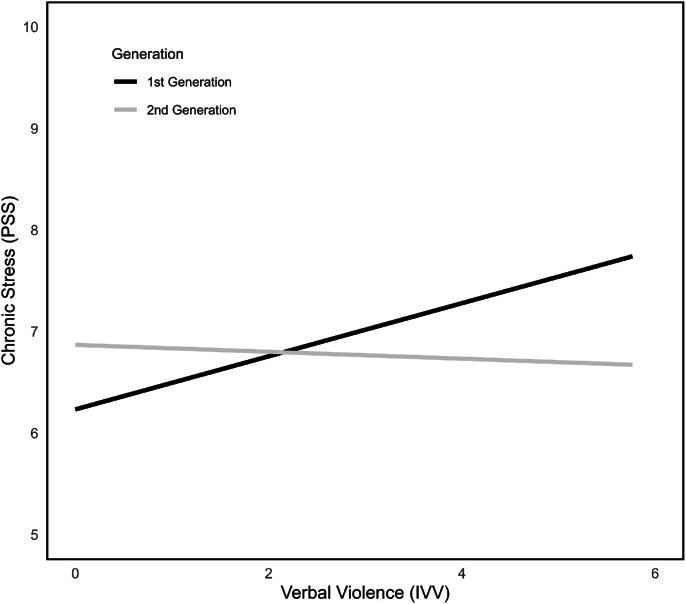
Interactions between IVV, PSS and first and second migration generation.

**Fig. 4:**
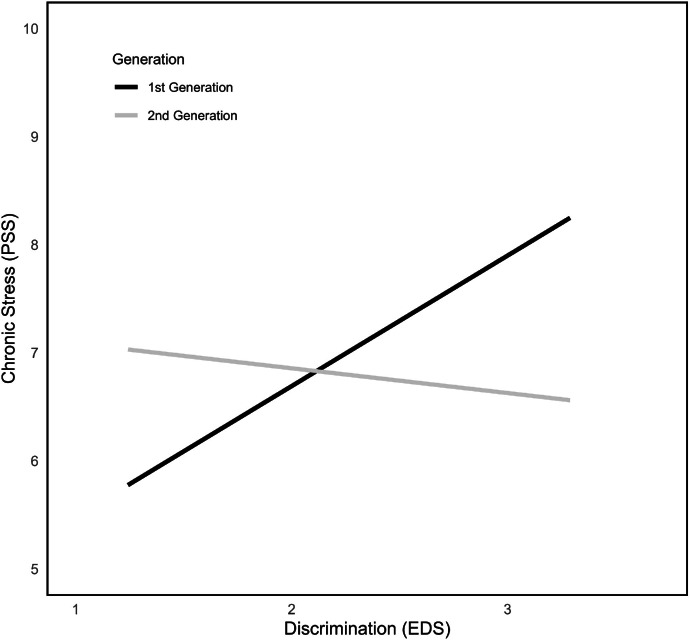
Interactions between EDS, PSS and first and second migration generation.

Examining the effect of migration generation, models with generation as moderator variable W were calculated. Results showed no significant overall interaction when IVV served as predictor variable X (n.s., model 5), but significant simple effects for the first generation only (*b* = 0.26, *SE* = 0.09, *p* < .01, 95 % CI [0.09; 0.43]; see Fig. 3). Looking at ethnic discrimination as predictor instead of IVV, the overall model (*F*(3228) = 10.40, *p* < .001) explained 12.11 % of variance (model 7). The moderation effect between ethnic discrimination and migration generation was significant, indicating that first-generation immigrants who reported higher values of chronic stress experience discrimination more often (ΔR² = 3.1 %, *F*(1228) = 5.52, *p* = .02; see Fig. 4). Second-generation immigrants did not report discrimination-dependent stress values, which could be an indicator for some form of protection when belonging to the second-generation group.

**Fig. 5:**
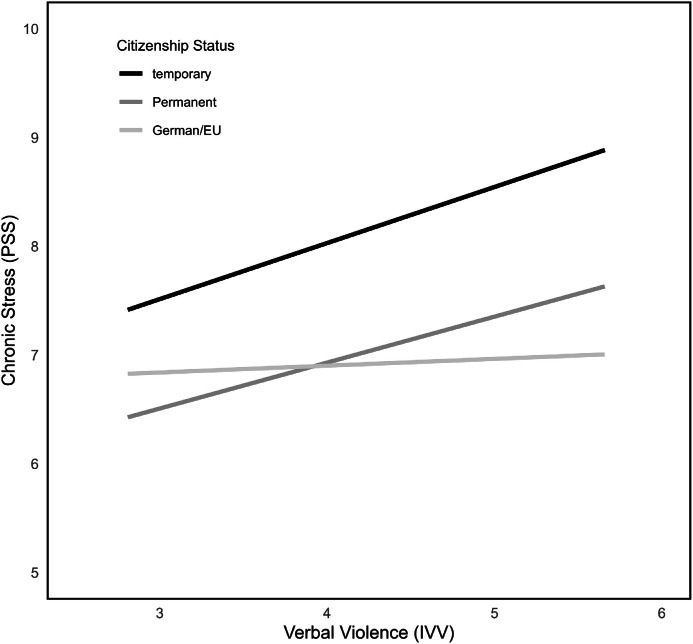
Interactions between IVV, PSS at different levels of citizenship status.

Examining the influence of citizenship status upon the connection between IVV and chronic stress perceptions, the overall model (*F*(5215) = 2.94, *p* = .01) explained 6.40 % of variance (model 6). The interaction term did not reach significance, but simple effects showed significant connections between IVV and chronic stress when looking at the temporary (*b* = 0.52, *SE* = 0.24, *p* = .03, 95 % CI [0.04; 0.99]) and the permanent residency group (*b* = 0.42, *SE* = 0.15, *p* < .01, 95 % CI [0.12; 0.72]; see Table A.1 in the Appendix and Fig. 5). Whereas persons with German and EU citizenship did not show this connection (n.s.), indicating not having citizenship status in another country or having the potentially stressful experience of applying for one might lead to more frequent experiences of IVV and therefore its consequences as well.

**Fig. 6:**
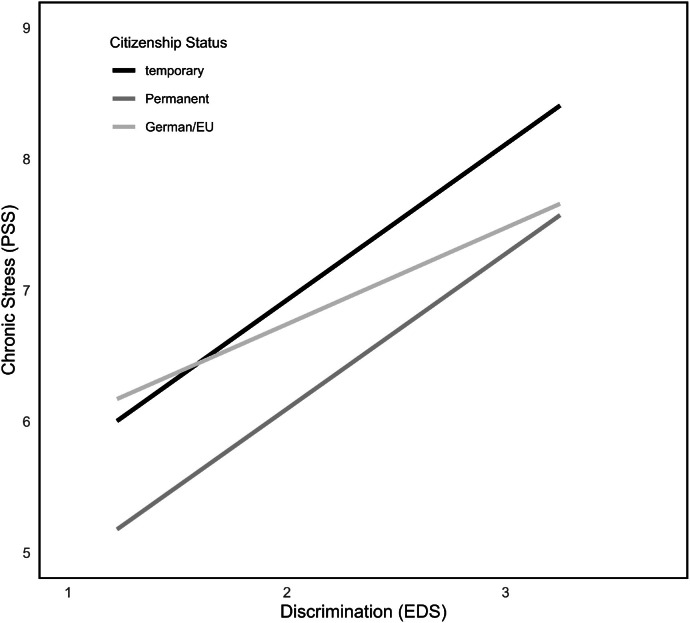
Interactions between EDS, PSS at different levels of citizenship status.

Looking at the model with ethnic discrimination as predictor variable X (model 8), results show an overall significant model (*F*(5215) = 4.06, *p* < .01, R^2^ = 0.09). The interaction term which tested potential moderation of citizenship status upon the connection between ethnic discrimination and chronic stress did not reach significance (n.s.). Still, comparably to IVV as predictor, significant conditional effects for the different categories of citizenship status got visible (temporal residency: *b* = 1.19, *SE* = 0.59, *p* = .047, 95 % CI [0.02; 2.36]; permanent residency: *b* = 1.18, *SE* = 0.42, *p* < .01, 95 % CI [0.36; 2.00]; German and EU citizenship: *b* = 0.73, *SE* = 0.34, *p* = .03, 95 % CI [0.07; 1.40]; see Table 3 and Fig. 6). Further values can be seen in Table 3.

### 3.5 Moderated moderation models

To combine migration-specific variables with protective factors, moderated moderation analyses (model 3 in PROCESS) were calculated. In the first step, immigration generation was used as variable Z, potentially moderating the protective factors resilience and self-compassion (variable W). All moderated moderation including resilience did not reach significance and are not further elaborated on. Specific results can be viewed in the Appendix (Table A.2 and A.3).

#### 3.5.1 Immigration generation and self-compassion

**Fig. 7 and 8:**
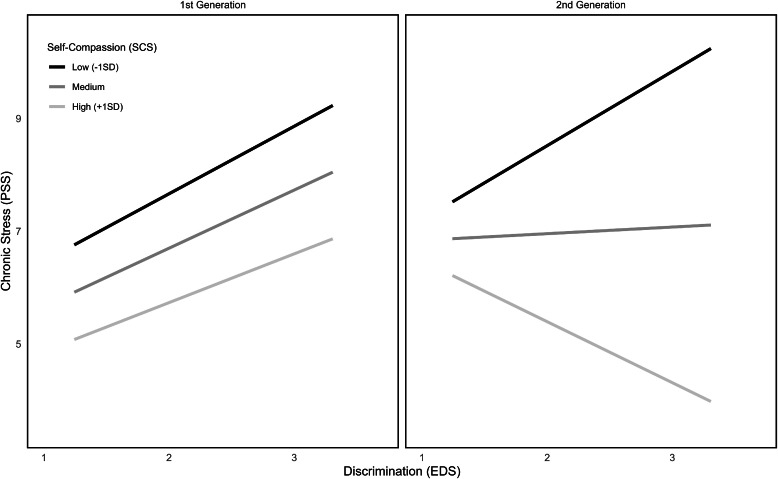
Moderated moderations between self-compassion, EDS, PSS and first and second migration generation.

When examining self-compassion as protective factor upon the connection between IVV and chronic stress, the overall model reached significance (*F*(7212) = 11.49, *p* < .001), explaining 22.8 % of variance. The triple interaction IVV x SCS x generation was significant (ΔR² = 2.2 %, *F*(1212) = 8.91, *p* < .01), as can be seen in Figs. 7,8. On closer examination of the conditional IVV x self-compassion interaction for first or second generation, only the second generation showed a significant interaction (*F*(1212) = 8.91, *p* < .01). These results indicate, that only in the second-generation subgroup, self-compassion serves as a protective factor against the connection between IVV and chronic stress. In this group, persons with high self-compassion experienced less IVV associated chronic stress whereas in the first-generation sub-group, participants experienced higher values of chronic stress when experiencing more IVV.

**Fig. 9 and 10:**
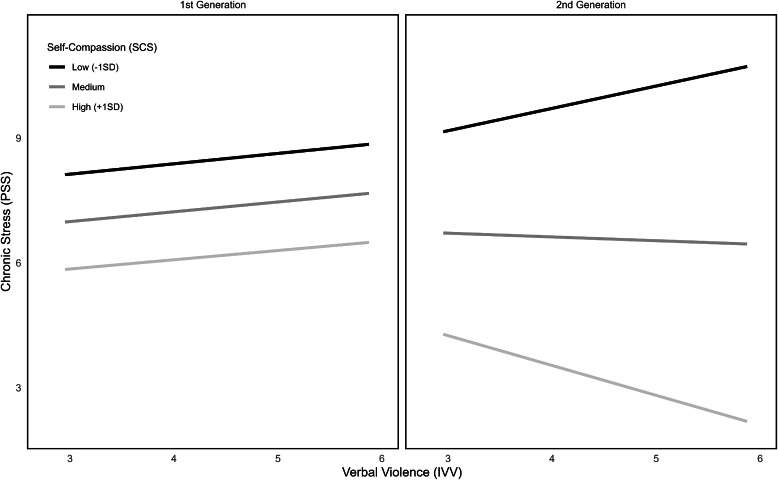
Moderated moderations between self-compassion, IVV, PSS and first and second migration generation.

When examining these relations for ethnic discrimination as predictor X, the overall model shows significance (*F*(7212) = 14.38, *p* < .001), explaining 28.1 % variance. The triple interaction between discrimination x SCS x generation was significant (ΔR² = 1.5 %, *F*(1212) = 6.61, *p* = .01; see Figs. 9,10). When looking at discrimination x SCS interactions for the two generations, only the second-generation group shows a significant interaction (*F*(1212) = 10.92, *p* < .01). Just as for IVV, higher levels of SCS seem to protect against discrimination based chronic stress only in the second-generation group.

#### 3.5.2 Citizenship status and self-compassion

**Fig. 11, 12 and 13:**
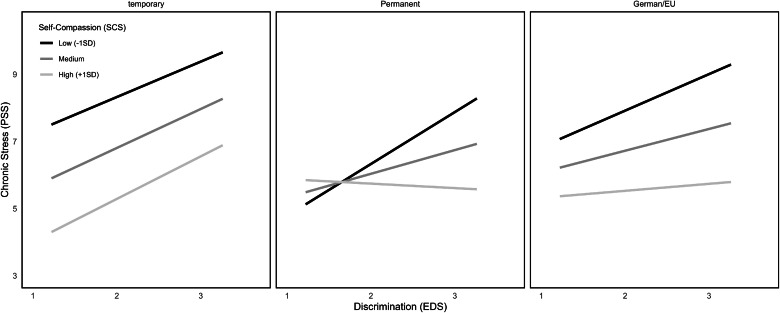
Moderated moderations between self-compassion, EDS, PSS and citizenship status.

When examining the influence of citizenship status (variable Z) in combination with self-compassion (variable W) and IVV (variable X), the overall model reached significance (*F*(11,198) = 6.54, *p* < .001, R^2^ = 0.23). Still, neither the triple interaction (n.s.), nor the conditional interactions for different citizenship status was significant (n.s.; see Figs. 11–13). These results indicate that the protective effect of self-compassion on the relationship between IVV and chronic stress does not depend on citizenship status.

**Fig. 14, 15 and 16:**
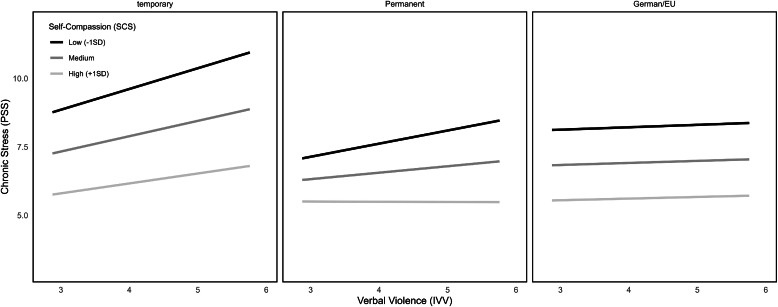
Moderated moderations between self-compassion, IVV, PSS and citizenship status.

Repeating these calculations for discrimination as variable X, the overall model reached significance (*F*(11,198) = 9.05, *p* < .001, R^2^ = 0.269). Comparably to the calculations above, the triple interactions did not reach significance (*p* > .05). Considering conditional discrimination x SCS interactions for different citizenship status, only the interaction for group 3 (German/EU citizenship) was significant (*F*(1198) = 3.99, *p* = .047). Considering this group solely, higher levels of self-compassion were able to buffer discrimination-associated stress (see Figs. 14–16).

## 4 Discussion

The present study examined the association of protective and risk factors, such as self-compassion, resilience and migration-specific variables, with discrimination, IVV and perceived chronic stress in an immigrant sample in Germany. Results showed that IVV as well as ethnic discrimination were robust predictors of perceived chronic stress. Still, including both predictors into a multiple regression model, only discrimination was able to explain further variance of chronic stress perception. Resilience alone did not act as a protective factor, whereas self-compassion had a protective effect on the association between discrimination and chronic stress. When including migration-specific variables, in particular being a first-generation immigrant moderated the relationship between chronic stress and discrimination. Those individuals reported higher levels of chronic stress the more ethnic discrimination they experienced. Moderated moderation analyses revealed that self-compassion appeared to be particularly protective for the second-generation subgroup, when experiencing IVV and discrimination, whereas no moderating effects of resilience were found. For citizenship status, a connection between IVV and chronic stress for temporary and permanent residency groups, but not for German/EU citizens, was found. In the moderated moderation, neither resilience nor self-compassion proved to be protective for the individual groups (temporary, permanent, EU/German). Examining only the EU/German citizenship group, an interaction between self-compassion and ethnic discrimination on chronic stress became apparent.

Results revealed, the more IVV or discrimination an individual reported, the more chronic stress was perceived. These findings are in line with our hypotheses and previous studies (Goreis et al., 2020; Mewes et al., 2015). In the case of IVV, the positive association with chronic stress as well as the high correlation with discrimination may be an indication of the similarity of the constructs and its comparable association with perceived stress. In the long term, chronic stress is known to predict adverse physical and mental health outcomes, as described by allostatic load (McEwen, 1998; Rohleder, 2019). For this reason, the relationships between stress-inducing and protective variables were examined.

Results revealed that resilience, against our hypotheses, was no moderating factor within this sample, neither for discrimination nor for IVV. Even though resilience was negatively associated to chronic stress perception, it did not moderate the association between ethnic discrimination or IVV and chronic stress. These findings are in line with a previous study (Cano et al., 2023), in which the authors noted that these null findings could be explained by the use of a rather short questionnaire, that may not have been able to capture the complexity of resilience. Current discussions scrutinize the informative value of resilience questionnaires in general and propagate a closer look at underlying mechanisms, such as flexible self-regulation, in longitudinal designs (Bonanno, 2021).

Self-compassion, however, moderated the connection between discrimination and chronic stress, but not between IVV and chronic stress. These results are in line with previous research that found buffering effects for self-compassion or its sub-facets on psychological distress or symptoms (Liu et al., 2020; Li et al., 2022; Schick et al., 2021). Self-compassion appears to be a sufficient emotion regulation strategy to reduce discrimination-related stress. Surprisingly, self-compassion did not moderate the connection between discrimination and IVV, leading to the question how and to what extent the constructs of discrimination and IVV differ in their harmful effects on health. We assume that the discrimination questionnaire includes a broader spectrum of discriminatory experiences by asking about everyday situations compared to the IVV questionnaire which refers to institutional situations only. It is conceivable that, while self-compassion may serve as mitigating factor against discriminatory incidents in everyday life, its efficacy does not extend to the potentially more existential significance of IVV scenarios.

Examining migration-related variables, generation moderated the association between discrimination and chronic stress. Especially first-generation immigrants dealt with discrimination-related stress, while individuals who grew up and were socialized in Germany did not show this connection. These results are in line with previous research on generation (Abdulhamed et al., 2022; Morawa et al., 2017; Beutel et al., 2016) and indicate that persons who undergo a certain migration and immigration process experience more negative health outcomes or discrimination related-stress. Further, they might already deal with several other stressors, such as acculturative stressors (e.g. learning a new language, being confronted with a new culture) (Berry et al., 1987), whereby they may be more vulnerable to the negative effects of discrimination. Regarding self-compassion as protective factor, the present study revealed a buffering effect only for individuals of the second-generation. Resilience was no protective factor, regardless of migration generation. This underscores the former described reasoning that self-compassion cannot unfold its potential for stressors which threaten a rather existential level (such as first-generation individuals may deal with in terms of right to stay, financial and medical support or work permit).

Considering citizenship status, discrimination seems to have an effect on chronic stress perceptions in every group (temporary, permanent, German/EU), whereas IVV only affected groups which somehow had to endure some forms of application process before (temporary and permanent). The group of individuals with permanent residency permit also reported the highest levels of discrimination. This could indicate that this subgroup may be sensitised to such experiences due to having a higher dependency on institutional decisions. Previous research with different immigrant groups in the U.S. showed that both naturalized as well as non-citizens reported poorer health compared to U.S.-born citizens (Bacong, 2021) or that non-citizens reported greater psychological distress compared to both other groups (Gee et al., 2016). Compared with our results, this could be an indicator that especially non-citizens form a vulnerable group considering psychological and physiological health.

The present study has several strengths and limitations. First, our study stands among the first to examine citizenship status and its associations with chronic stress in Germany. A stronger focus upon structural factors as well as immigrant policies was named to be significant when examining immigrants’ health in previous research (Viruell-Fuentes et al., 2012). Second, German research focussing on protective factors in immigrant samples is scarce. Whereas its relevance is underscored by developments over the past decade, culminating in Germany becoming the country with the second-highest number of immigrants worldwide (International Organization for Migration, 2021). Third, by providing our online survey in several languages, we aimed to facilitate participation from populations that might have been otherwise challenging to reach due to language barriers.

Several limitations need to be noted. Even though the heterogeneity of our study sample captures a diverse picture of migration-specific burdens, it leads to neglecting differences between subpopulations (e.g. specific countries of origin or reasons to migrate). This is particularly relevant as different groups experience discrimination in different ways (Beigang et al., 2017; Gong et al., 2017). Besides, equally analysing questionnaires used in different languages and populations may underestimate cultural and linguistic influences upon the found associations. In addition, our Verbal Violence Questionnaire exhibited a narrow range of responses, leading to limited significant correlations due to reduced variance. This underscores the need for further research regarding this construct or a revision for which samples it might be suitable. Further, we emphasize that including multiple explanatory variables in our moderation models results in small subgroups, which consequently leads to underpowering of our analyses. Future research should consider larger sample sizes to ensure representative subgroups.

Nevertheless, our study was the first to focus on and quantify IVV and its consequences for migrants in Germany. Further analyses, including psychometric analyses, are necessary to clearly differentiate our operationalisation of IVV from the concept of discrimination. The present study shows that, even though both constructs appear to explain a high amount of shared variance, the concepts differed in their influence on the outcome for certain values of the moderators. Additionally, future research might focus on and compare certain institutional settings and their inherent stress exacerbating dispositions for immigrants and their acculturation process. Moreover, longitudinal research in experimental settings might give further insights on causal relationships and should include physiological markers, to be able to make predictions about health outcomes.

Practically, our findings underscore the implementation of tailored interventions (Cano et al., 2023), as discrimination will probably never be completely eliminated. These could include, as already implemented in acute stress paradigms (Arch et al., 2014), self-compassion trainings, to strengthen vulnerable groups from discrimination-related stress. Furthermore, in existing therapeutic programs, such as mindfulness-based stress reduction (MBSR) (Kabat-Zinn, 2003), self-compassion components could be integrated and these programs tailored to specific immigrant groups. Cultural sensitivity and inclusion are crucial in these adaptions, extending to the training of service providers in both healthcare and public authorities. These trainings should emphasize cultural sensitivity, highlight the adverse consequences of discrimination and verbal violence, focus on recognizing signs of stress, and provide brief self-compassion strategies. Additionally, these findings should inform public policies, leading to the integration of self-compassion strategies into public health initiatives to better support immigrant communities. However, these findings on self-compassion as a protective factor should not overshadow the examined stress-generating outcomes at a structural level, such as citizenship status, which are inherently difficult to change. It is crucial to emphasize that structural changes to reduce immigrants’ stressors need to be reconsidered on a societal level, to ensure they are not held solely responsible for adverse health outcomes, for example, through participation in training programs.

## 5 Conclusions

In summary, this study emphasizes the disadvantageous associations of experiencing IVV and discrimination, whether in institutional contexts or in daily life for an immigrant sample in Germany. Besides, self-compassion as protective factor might be implemented in interventions for immigrant groups who suffer from discrimination, but who experience a certain level of security in terms of residence status and social and societal participation. Particularly vulnerable groups, such as first-generation migrants and individuals who are not naturalised and have no citizenship, need to be examined more closely in further research.

## Funding

This work was funded by the Emerging-Fields-Initiative (EFI) of the Friedrich-Alexander-University Erlangen-Nürnberg, the STAEDTLER-Stiftung, and the 10.13039/501100001659Deutsche Forschungsgemeinschaft (DFG; German Research Foundation) - SFB 1483 - Project-ID 442419336, EmpkinS, and Project-ID 523958832.

## Ethical approval

This study was performed in line with the principles of the Declaration of Helsinki. Approval was granted by the Ethics Committee of the Medical Faculty of the Friedrich-Alexander-Universität Erlangen-Nürnberg (ethical approval code: 451_19B).

## Consent to participate

Informed consent was obtained from all individual participants included in the study.

## CRediT authorship contribution statement

**Felicitas Hauck:** Writing – review & editing, Writing – original draft, Visualization, Software, Methodology, Investigation, Formal analysis, Data curation, Conceptualization. **Andrea Borho:** Writing – review & editing, Software, Methodology, Investigation, Data curation, Conceptualization. **Lucía Romero Gibu:** Methodology, Data curation, Conceptualization. **Mojib Atal:** Methodology, Investigation, Data curation, Conceptualization. **Sevil Dederer:** Methodology, Investigation, Conceptualization. **Petra Bendel:** Project administration, Methodology, Funding acquisition, Conceptualization. **Eva Morawa:** Project administration, Methodology, Investigation, Funding acquisition, Conceptualization. **Yesim Erim:** Project administration, Methodology, Funding acquisition, Conceptualization. **Silke Jansen:** Project administration, Methodology, Funding acquisition, Conceptualization. **Nicolas Rohleder:** Writing – review & editing, Visualization, Supervision, Project administration, Methodology, Funding acquisition, Formal analysis, Data curation, Conceptualization.

## Declaration of competing interest

The authors declare that they have no known competing financial interests or personal relationships that could have appeared to influence the work reported in this paper.

## Appendix Supplementary materials

Image, application 1
