# Supplementary material for: The association of perceived ethnic discrimination and institutional verbal violence with chronic stress in an immigrant sample: The role of protective factors - results from the VIOLIN study
Source: J Migr Health. 2024 Aug 4;10:100260. doi: 10.1016/j.jmh.2024.100260 (PMC11365374; doi:10.1016/j.jmh.2024.100260)
Supplement: Supplementary file 1 [file mmc1.docx]

# Appendix

## Group differences for migration-specific variables

In a first step, we checked for differences of IVV and discrimination frequencies as well as chronic stress perceptions between the first or second generation groups. Differences were tested with Man-Whitney-U-tests for independent samples. No significant differences were found (n.s.). Further, these differences were tested for citizenship status as well, using a one-way MANOVA with citizenship status as between factor. The MANOVA found no statistically significant differences between citizenship status on the combined dependent variables, *F*(6,432) = 1.66, *p* = .13, partial η² = .023. Post-hoc univariate ANOVAs were conducted for IVV, discrimination and chronic stress and showed a significant difference between the citizenship status for experienced ethnic discrimination, *F*(2, 218) = 3.34,
*p* = .04, partial η² = .03, but neither for IVV (*F*(2, 218) = 1.40, *p* = .25, partial η² = .013), nor for chronic stress (*F*(2, 218) = 0.61, *p* = .54, partial η² = .006; see Figure 3). Tukey HSD post-hoc analysis on ethnic discrimination revealed the tendency of a significant difference between the group of German/EU-citizens (*M* = 2.1, *SD* = 1.01) and persons with permanent residency (*M* = 2.52, *SD* = 1.18; MDiff = 0.4155, p = .05, 95% CI[-0.01, 0.84]), but no significant differences between the other groups (n.s.; for temporary: *M* = 2.39; *SD* = 0.89).


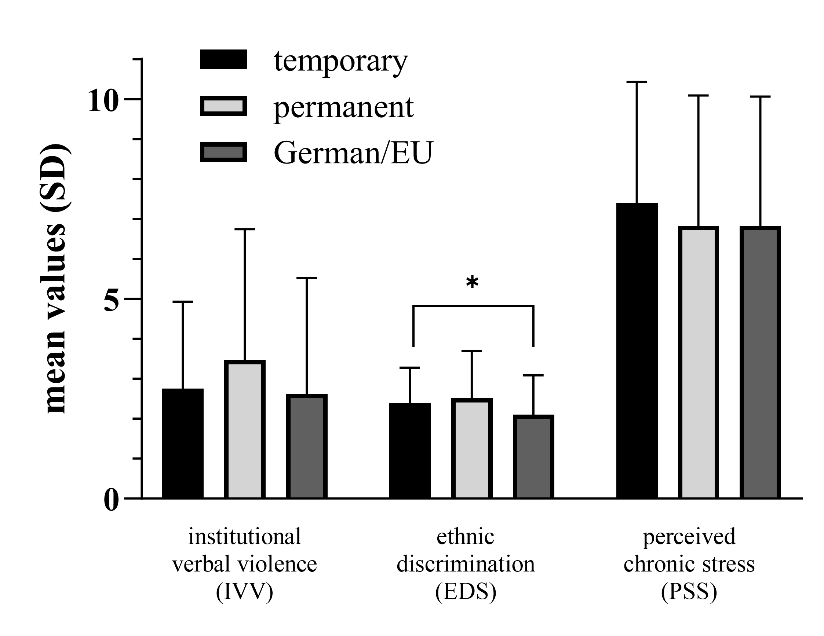


**Figure A.1** Differences for citizenship status in IVV, EDS and PSS, * p < .05

## Conditional, simple effects

**Table A.1** Conditional, simple effects of IVV and EDS at different citizenship status values.

| **Predictor (X)** | **Moderator (W) Citizenship Status** | **Effect** | **SE** | ***t*** | ***p*** | **95% CI** | |
| --- | --- | --- | --- | --- | --- | --- | --- |
|  |  |  |  |  |  | **Lower** | **Upper** |
| **Model 7 (citizenship status and IVV)** | | | | | | | |
| IVV | temporary | 0.52 | 0.24 | 2.15 | .03 | 0.04 | 0.99 |
|  | permanent | 0.42 | 0.15 | 2.77 | .01 | 0.12 | 0.72 |
|  | EU/German Status | 0.06 | 0.12 | 0.52 | .60 | -0.17 | 0.30 |
| **Model 8 (citizenship status and EDS)** | | | | | | | |
| EDS | temporary | 1.19 | 0.59 | 2.00 | .047 | 0.02 | 2.36 |
|  | permanent | 1.18 | 0.42 | 2.83 | .01 | 0.36 | 2.00 |
|  | EU/German Status | 0.73 | 0.34 | 2.17 | .03 | 0.07 | 1.40 |

*Note.* SE = Standard Error; t = t-Value; p = p-Value; 95% CI = Confidence Interval; IVV = Institutional Verbal Violence total score; EDS = Everyday Discrimination Scale; Status = Citizenship Status (1 = temporary, 2 = permanent; 3 = German or EU citizenship).

## Moderated moderation models

**Table A.2** Moderated moderation analyses with protective variables as moderators, immigration generation as moderating moderator and PSS as outcome.

|  | **Coefficient** | **SE** | ***t*** | ***p*** | **95% CI** | |
| --- | --- | --- | --- | --- | --- | --- |
|  |  |  |  |  | **Lower** | **Upper** |
| **Model 9 (generation, SCS, IVV)** | | | | | | |
| Generation | -1.77 | 3.46 | -0.51 | .61 | -8.59 | 5.04 |
| SCS | -2.41 | 1.40 | -1.72 | .09 | -5.18 | 0.35 |
| IVV | -2.31 | 1.23 | -1.87 | .06 | -4.74 | 0.12 |
| IVV x SCS | 0.90 | 0.37 | 2.40 | .02 | 0.16 | 1.63 |
| Gen x SCS | 0.77 | 1.02 | 0.75 | .45 | -1.25 | 2.79 |
| Gen x IVV | 2.61 | 1.01 | 2.60 | .01 | 0.63 | 4.59 |
| Gen x IVV x SCS | -0.92 | 0.31 | -2.99 | .003 | -1.52 | -0.31 |
| **Model 10 (generation, SCS, EDS)** | | | | | | |
| Generation | -4.95 | 5.12 | -0.97 | .33 | -15.05 | 5.14 |
| SCS | -3.12 | 2.04 | -1.54 | .12 | -7.16 | 0.88 |
| EDS | -2.20 | 2.38 | -0.92 | .36 | -6.90 | 2.50 |
| EDS x SCS | 1.30 | 0.75 | 1.73 | .09 | -0.18 | 2.77 |
| Gen x SCS | 2.20 | 1.63 | 1.35 | .18 | -1.01 | 5.41 |
| Gen x EDS | 4.03 | 1.91 | 2.11 | .036 | 0.27 | 7.79 |
| Gen x EDS x SCS | -1.54 | 0.60 | -2.57 | .011 | -2.73 | -0.36 |
| **Model 11 (generation, RS, IVV)** | | | | | | |
| Generation | -3.34 | 3.04 | -1.10 | .27 | -9.34 | 2.66 |
| RS | -0.14 | 0.08 | -1.77 | .08 | -0.30 | 0.02 |
| IVV | -0.01 | 1.79 | -0.004 | .997 | -3.54 | 3.52 |
| IVV x RS | 0.01 | 0.03 | 0.26 | .79 | -0.05 | 0.06 |
| Gen x RS | 0.06 | 0.05 | 1.16 | .25 | -0.04 | 0.16 |
| Gen x IVV | 0.54 | 1.61 | 0.34 | .74 | -2.63 | 3.71 |
| Gen x IVV x RS | -0.01 | 0.02 | -0.48 | .63 | -0.06 | 0.04 |
| **Model 12 (generation, RS, EDS)** | | | | | | |
| Generation | -3.37 | 4.75 | -0.71 | .48 | -12.72 | 5.99 |
| RS | -0.16 | 0.11 | -1.43 | .15 | -0.37 | 0.06 |
| EDS | 0.47 | 3.38 | 0.14 | .89 | -6.19 | 7.14 |
| EDS x RS | 0.03 | 0.06 | 0.47 | .64 | -0.08 | 0.13 |
| Gen x RS | 0.09 | 0.08 | 1.07 | .29 | -0.07 | 0.25 |
| Gen x EDS | 0.89 | 2.92 | 0.31 | .76 | -4.86 | 6.64 |
| Gen x EDS x RS | -0.03 | 0.05 | -0.65 | .52 | -0.12 | 0.06 |

*Note.* SE = Standard Error; t = t-Value; p = p-Value; 95% CI = Confidence Interval; Gen = Immigration Generation; IVV = Institutional Verbal Violence total score; EDS = Everyday Discrimination Scale; PSS = Perceived Stress Scale.

**Table A.3** Moderated moderation analyses with protective variables as moderators, citizenship status as moderating moderator and PSS as outcome.

|  | **Coefficient** | **SE** | ***t*** | ***p*** | **95% CI** | |
| --- | --- | --- | --- | --- | --- | --- |
|  |  |  |  |  | **Lower** | **Upper** |
| **Model 13 (status, SCS, IVV)** | | | | | | |
| Status Z1 | -4.11 | 5.95 | -0.69 | .49 | -15.85 | 7.63 |
| Status Z2 | 2.46 | 4.52 | 0.54 | .59 | -6.46 | 11.37 |
| SCS | -1.40 | 1.26 | -1.11 | .27 | -3.87 | 1.08 |
| IVV | 1.51 | 1.03 | 1.47 | .14 | -0.52 | 3.54 |
| IVV x SCS | -0.30 | 0.31 | -0.95 | .34 | -0.91 | 0.32 |
| Stat Z1 x SCS | 1.27 | 1.81 | 0.70 | .48 | -2.29 | 4.83 |
| Stat Z2 x SCS | -0.46 | 1.39 | -0.33 | .74 | -3.20 | 2.28 |
| Stat Z1 x IVV | -0.10 | 1.21 | -0.08 | .93 | -2.48 | 2.28 |
| Stat Z2 x IVV | -1.37 | 1.18 | -1.15 | .25 | -3.70 | 0.97 |
| Stat Z1 x IVV x SCS | -0.07 | 0.37 | -0.19 | .85 | -0.81 | 0.67 |
| Stat Z2 x IVV x SCS | 0.27 | 0.35 | 0.78 | .44 | -0.42 | 0.97 |
| **Model 14 (status, SCS, EDS)** | | | | | | |
| Status Z1 | -14.83 | 10.25 | -1.45 | .15 | -35.04 | 5.38 |
| Status Z2 | -5.86 | 6.80 | -0.86 | .39 | -19.27 | 7.56 |
| SCS | -2.59 | 1.95 | -1.32 | .19 | -6.44 | 1.27 |
| EDS | 0.65 | 2.59 | 0.25 | .80 | -4.46 | 5.76 |
| EDS x SCS | 0.16 | 0.77 | 0.21 | .84 | -1.35 | 1.67 |
| Stat Z1 x SCS | 4.66 | 3.12 | 1.49 | .14 | -1.50 | 10.82 |
| Stat Z2 x SCS | 2.12 | 2.09 | 1.01 | .31 | -2.01 | 6.24 |
| Stat Z1 x EDS | 4.09 | 3.31 | 1.24 | .22 | -2.43 | 10.61 |
| Stat Z2 x EDS | 2.11 | 2.77 | 0.76 | .45 | -3.36 | 7.58 |
| Stat Z1 x EDS x SCS | -1.41 | 1.01 | -1.40 | .16 | -3.40 | 0.58 |
| Stat Z2 x EDS x SCS | .0.82 | 0.83 | -0.98 | .33 | -2.46 | 0.83 |
| **Model 15 (status, RS, IVV)** | | | | | | |
| Status Z1 | 1.61 | 4.63 | 0.35 | .73 | -.7.52 | 10.74 |
| Status Z2 | 2.35 | 3.78 | 0.62 | .53 | -5.10 | 9.81 |
| RS | -0.06 | 0.05 | -1.25 | .21 | -0.16 | 1.96 |
| IVV | 0.16 | 0.91 | 0.17 | .86 | -1.65 | 1.96 |
| IVV x RS | 0.01 | 0.02 | 0.41 | .68 | -0.03 | 0.04 |
| Stat Z1 x RS | -0.03 | 0.08 | -0.36 | .72 | -0.19 | 0.13 |
| Stat Z2 x RS | -0.02 | 0.06 | -0.32 | .75 | -0.15 | 0.11 |
| Stat Z1 x IVV | 0.57 | 1.09 | 0.53 | .60 | -1.58 | 2.72 |
| Stat Z2 x IVV | 0.42 | 1.14 | 0.37 | .71 | -1.94 | 2.67 |
| Stat Z1 x IVV x RS | -0.01 | 0.02 | -0.65 | .52 | -0.05 | 0.02 |
| Stat Z2 x IVV x RS | -0.01 | 0.02 | -0.74 | .46 | -0.05 | 0.02 |
| **Model 16 (status, RS, EDS)** | | | | | | |
| Status Z1 | 0.62 | 7.19 | 0.09 | .93 | -13.56 | 14.79 |
| Status Z2 | -0.74 | 5.20 | -0.14 | .89 | -10.99 | 9.51 |
| RS | -0.09 | 0.09 | -1.01 | .31 | 0.26 | 0.08 |
| EDS | 0.37 | 2.29 | 0.16 | .87 | -4.16 | 4.89 |
| EDS x RS | 0.02 | 0.04 | 0.38 | .70 | -0.07 | 0.10 |
| Stat Z1 x RS | -0.01 | 0.13 | -0.04 | .97 | -0.26 | 0.25 |
| Stat Z2 x RS | 0.04 | 0.10 | 0.41 | .68 | -0.15 | 0.23 |
| Stat Z1 x EDS | 1.02 | 3.12 | 0.33 | .74 | -5.12 | 7.17 |
| Stat Z2 x EDS | 1.51 | 2.62 | 0.58 | .56 | -3.65 | 6.67 |
| Stat Z1 x EDS x RS | -0.02 | 0.05 | -0.40 | .69 | .0.13 | 0.08 |
| Stat Z2 x EDS x RS | -0.04 | 0.05 | -0.77 | .44 | -0.13 | 0.06 |

*Note.* SE = Standard Error; t = t-Value; p = p-Value; 95% CI = Confidence Interval; IVV = Institutional Verbal Violence total score; EDS = Everyday Discrimination Scale; Status/Stat = Citizenship Status (1 = temporary, 2 = permanent; 3 = German or EU citizenship); Z1 = moderating Moderator 1 (Coded categorical variable Z with 1 = 0; 2 = 1; 3 = 0); Z2 = moderating Moderator 2 (coded with 1 = 0; 2 = 0; 3 = 1); PSS = Perceived Stress Scale.
